# Environmental Enrichment Alters Nicotine-Mediated Locomotor Sensitization and Phosphorylation of DARPP-32 and CREB in Rat Prefrontal Cortex

**DOI:** 10.1371/journal.pone.0044149

**Published:** 2012-08-31

**Authors:** Adrian M. Gomez, Narasimha M. Midde, Charles F. Mactutus, Rosemarie M. Booze, Jun Zhu

**Affiliations:** 1 Department of Pharmaceutical and Biomedical Sciences, South Carolina College of Pharmacy, University of South Carolina, Columbia, South Carolina, United States of America; 2 Department of Psychology, University of South Carolina, Columbia, South Carolina, United States of America; INSERM/CNRS, France

## Abstract

Exposure within an environmental enrichment paradigm results in neurobiological adaptations and decreases the baseline of locomotor activity. The current study determined activation of DARPP-32 (dopamine- and cAMP-regulated phosphoprotein-32) and CREB (cAMP response element binding protein), and locomotor activity in rats raised in enriched (EC), impoverished (IC), and standard (SC) conditions following repeated administration of nicotine or saline. In the saline-control group, the basal phosphorylation state of DARPP-32 at Threonine-34 site (pDARPP-32 Thr34) in the prefrontal cortex (PFC) was lower in EC compared to IC and SC rats, which was positively correlated with their respective baseline activities. While nicotine (0.35 mg/kg, freebase) produced locomotor sensitization across all housing conditions when the nicotine-mediated locomotor activity was expressed as a percent change from their respective saline control, EC rats displayed greater sensitization to nicotine than IC and SC rats. Consistent with the behavioral findings, repeated nicotine injection increased pDARPP-32 Thr34 in PFC of EC and IC rats and in nucleus accumbens of EC rats; however, the magnitude of change from saline control in nicotine-induced enhancement of pDARPP-32 Thr34 in PFC was strikingly increased in EC rats relative to IC rats. Moreover, EC rats had lower basal phosphorylation levels of CREB at serine 133 in PFC and nucleus accumbens compared to IC and SC rats, whereas the nicotine-induced increase in phosphorylated CREB-Ser133 was more pronounced in PFC of EC rats relative to IC and SC rats. Collectively, these findings suggest innovative insights into advancing our understanding of the molecular mechanisms of enrichment-induced changes in the motivational effects of nicotine, and aiding in the identification of new therapeutic strategies for tobacco smokers.

## Introduction

Convergent evidence suggests that environmental factors impact individual susceptibility to drugs of abuse [1], [2]. One animal model that addresses environmental factors uses rats raised in one of the three different conditions: an enriched condition (EC), a standard condition (SC), and an impoverished condition (IC). This animal model provides an ideal approach in elucidating the underlying neurobiological mechanisms of environmental influences on vulnerability to nicotine addiction [3].

Nicotine activates nicotinic acetylcholine receptors throughout the brain, thereby stimulating dopamine (DA) release within the mesocorticolimbic system [4]–[8]. Repeated exposure to nicotine induces behavioral sensitization [9]–[12]. Although behavioral sensitization is not a measure of drug reward, this procedure is sensitive to behavioral changes produced by the psychostimulant effects of abused drugs [13]–[15]. This procedure was used in the current study to determine whether enriched environment-induced alterations in locomotor sensitization to nicotine was associated with changes in dopaminergic signaling proteins. EC rats exhibit a reduction in nicotine-mediated locomotor activity compared to IC and SC rats [16], which could be mediated by enriched environment-induced alterations of dopaminergic pathways. Indeed, drug-naïve EC rats exhibit diminished DA transporter function [17], less synaptic DA levels in medial prefrontal cortex [18], [19], and show decreased D1 receptor function and expression in the prefrontal cortex (PFC) compared with IC and SC groups [20]. In contrast, repeated nicotine administration profoundly increases DA clearance and 3,4-Dihydroxyphenylacetic acid (DOPAC) levels in the PFC of EC rats but not in IC rats [21]. Therefore, EC rats may have lower dopaminergic tone compared to IC rats under basal conditions, which may contribute to differential behavioral responses to psychostimulants.

Activation of the DA/D1 receptor/cAMP/protein kinase A (PKA) pathway increases phosphorylation of DA and cAMP-regulated phosphoprotein-32 (DARPP-32) at the site Threonine 34 (pDARPP-32 Thr34), but decreases phosphorylation of DARPP-32 at Threonine 75 (pDARPP-32 Thr75) [22]. In contrast, phosphorylation of DARPP-32 at Thr75 by cyclin-dependent kinase 5 has a major inhibitory effect on the pDARPP-32 Thr34 by PKA, thereby reducing D1 DA signaling through the DARPP-32/ protein phosphatase 1 (PP-1) cascade [22], [23]. In addition, activation of the PKA pathway enhances phosphorylation of cAMP response element binding protein (CREB) at serine 133, which is essential for neuronal plasticity in response to repeated exposure to drugs [24]–[26]. Since phospho-Ser133 is dephosphorylated by PP-1, DARPP-32 also acts as a multifunctional protein that can modulate CREB phosphorylation [24], [27]. On the other hand, activation of Ca^2+^-dependent calcineurin by D2 receptors results in dephosphorylation of pDARPP-32 Thr34 [28]. Since nicotine-induced increased pDARPP-32 Thr34 and nicotine-mediated behavioral sensitization were attenuated by inhibition of calcineurin [29], we propose that environmental enrichment alters activation of DARPP-32 and CREB, and that these neuroadaptations may be associated with differential regulation of nicotine-mediated locomotor activity.

## Materials and Methods

### Ethics Statement

All of the experimental procedures in the animals were performed according to the National Institute of Health guidelines in AAALAC accredited facilities. The experimental protocol for this study was approved by the Institutional Animal Care and Use Committee (IACUC) at the University of South Carolina under compliance with animal welfare assurance #A3049-01.

### Subjects

Male Sprague-Dawley rats were obtained from Harlan Laboratories, Inc. (Indianapolis, IN). Rats arrived at the age of 21 days and were housed with food and water *ad libitum* in a colony room in the Division of Laboratory Animal Resources at the University of South Carolina, which was maintained at 21±2°C, 50±10% relative humidity and on a 12-h light/dark cycle with lights on at 07:00 AM.

### Environmental Conditions

Upon arrival, rats were assigned randomly to the EC, IC, or SC group using a previously published method [30]. EC rats were group-housed (10–15 per cage) in a metal cage (120 cm length×60 cm width×45 cm height). Twelve hard non-chewable plastic objects were placed randomly in the cage. EC rats were handled each day. Each day, half of the objects were replaced with new objects, the remaining objects were rearranged to boost novelty. IC rats were housed individually in wire mesh hanging cages (25 cm length×18 cm width×17 cm height) with solid metal sides and wire mesh floor. SC rats were pair-housed in a clear polycarbonate cage (43 cm×20 cm width×20 cm height) with wire rack top. IC and SC rats were neither handled nor exposed to any object except food and water. The SC condition conforms to the typical housing conditions set in the NIH Guide for the 1996 version of the NIH *Guide for the Care and Use of Laboratory Animals.* Rats were maintained in these environments until 53 days of age and throughout all experiments.

### Behavioral Apparatus

The activity apparatuses were square (40×40 cm) chambers (Hamilton-Kinder Inc., Poway, CA) that detect free movement of animals by infrared photocell interruptions. This equipment contained an infrared photocell grid (32 emitter/detector pairs) to measure locomotor activity with Digipro System Software (v.140, AccuScan Instruments). Each beam was spaced 2.5 cm apart and 7.0 cm above the chamber floor. The chambers were converted into round (∼40 cm diameter) compartments by adding clear Plexiglas inserts; photocell emitter/detector pairs were tuned by the manufacturer to handle the extra perspex width. Total horizontal activity measured all beam breaks in the horizontal plane with total rearing activity measuring all beam breaks in the vertical plane. All activity monitors were located in an isolated room away from the colony.

### Nicotine Administration

An initial experiment was performed to assess the dose effects of acute nicotine on DARPP-32 phosphorylation in brain regions of EC and IC rats. EC and IC rats were administered saline (1 ml/kg) or nicotine (0.1, 0.3, or 0.8 mg/kg) subcutaneously (s.c.). To determine whether enrichment results in differential changes in activation of DARPP-32 and CREB, and their behavioral response to repeated nicotine administration, an optimal dose of nicotine (0.35 mg/kg) was chosen based on results showing the dose effects of acute nicotine on DARPP-32 activity and the previous report demonstrating that nicotine at a similar dose (0.4 mg/kg) produces locomotor sensitization in both EC and IC rats [31]. Thus, in a subsequent experiment, rats from the EC, IC, and SC groups were randomly assigned to treatment groups and administered saline or nicotine (0.35 mg/kg, freebase, s.c.) daily for a total of 15 days. Nicotine was injected in a volume of 1 ml/kg body weight. Nicotine hydrogen tartrate salt was purchased from Sigma-Aldrich (St. Louis, MO) and dissolved in sterile saline (0.9% sodium chloride). The nicotine solution (freebase) was prepared immediately prior to injection and neutralized to pH 7.0 with NaHCO_3_ to reduce irritation.

### Habituation and Saline Baseline

All animals were habituated to the locomotor activity chambers for two 60-min sessions, once/day with no injection. Twenty-four hours after the second habituation session, all rats were habituated to the locomotor chambers for 30 min prior to injection, and then injected subcutaneously with saline and placed into the activity chambers for 60-min to measure baseline activity.

### Pre-injection Habituation and Nicotine-induced Locomotor Activity

The behavioral sensitization procedure began 24 h after the saline baseline measurement. All rats received a 30-min habituation period in the testing chamber prior to nicotine (0.35 mg/kg) or saline injection as reported previously [32]. This was done so that the onset of nicotine's effects did not overlap with the period that rats showed the most exploratory behavior in the chamber, which was during the first 15 min [33], [34]. After the 30-min habituation session, rats were administered nicotine (0.35 mg/kg) or saline. Subsequently, horizontal and rearing activities were assessed during the following 60-min session. Rats received saline or nicotine injections once/day for a total of 15 days; however, locomotor activities with regard to 30-min pre-injection session and 60-min post-injection session were recorded every other day, i.e., on days 1, 3, 5, 7, 9, 11, 13, and 15. During the “off” days of locomotor testing, rats were still transported to the same room where rats were injected for locomotor testing and then returned to home cages after nicotine or saline injection.

### Western Blot Analysis

Brains from rats acutely or repeatedly treated with nicotine were removed by rapid decapitation 20 min after the last injection. Brains were dissected in a chilled matrix. PFC, nucleus accumbens (NAc) and striatum were sonicated immediately on ice in a homogenization buffer containing 20 mM HEPES, 0.5 mM EDTA, 0.1 mM EGTA, 0.4 M NaCI, 5 mM MgCI2, 20% glycerol, 1 mM PMSF, phosphatase inhibitor cocktails I (P2850, Sigma-Aldrich, St. Louis, MO) and protease inhibitors (P8340, Sigma-Aldrich, St. Louis, MO), as described previously [32]. Samples were centrifuged at 12,000 g for 15 min. The supernatant was collected and stored at −80°C. Protein concentrations were determined in triplicate using Bio-Rad DC protein detection reagent. Proteins (60, 40, or 10 µg per sample in the PFC, NAc or striatum) were loaded for total DARPP-32, pDARPP-32 Thr34, pDARPP-32 Thr75, CREB, and phosphorylated CREB (pCREB, phosphor Ser133).

Proteins were separated by 10% SDS-polyacrylamide gel electrophoresis (SDS-PAGE) for 55–65 min at 150 V, and transferred to Immobilon-P transfer membranes (Cat # IPVH00010, 0.45 µm pore size; Millipore Co., Bedford, MA) in transfer buffer (50 mM Tris, 250 mM glycine, 3.5 mM SDS with 20% methanol) using a Mini Trans-Blot Electrophoretic Transfer Cell (Bio-Rad Laboratories Ltd., Hercules, CA) for 110 min at 75 V. Transfer membranes were then incubated with blocking buffer (5% dry milk powder in PBS containing 0.5% Tween 20) for 1 h at room temperature and then incubated overnight at 4°C in blocking buffer with primary antibodies against the following proteins: DARPP32 (1∶2000, 374-DARPP, PhosphoSolutions, Aurora, CO), pDARPP32 Thr34 (1∶1000, p1025–34, PhosphoSolutions, Aurora, CO), pDARPP32 Thr75 (1∶1000, p1025–75, PhosphoSolutions, Aurora, CO), CREB (1∶1000, 9104, Cell Signaling, Danvers, MA), pCREB (1∶1000, 9196L, Cell Signaling, Danvers, MA), respectively.

Blots were washed 10 min×3 times with wash buffer (PBS containing 0.5% Tween 20) at room temperature, and then incubated for 1 h in blocking buffer including one of the following secondary affinity-purified, horseradish peroxidase-conjugated antibodies: anti-rabbit IgG (111-035-144, Jackson ImmunoResearch, West Grove, PA; 1∶20000 for Total DARPP-32, 1∶7500 for pDARPP-Thr34 and pDARPP-Thr75), anti-mouse IgG (7076, Cell Signaling, Danvers, MA; 1∶2000 for both Total CREB and pCREB). Blots were then washed 3 times for 10 min per wash. Blots on the transfer membranes were detected using enhanced chemiluminescence (ECL-plus) and developed on Hyperfilm (Amersham Biosciences UK Ltd., Little Chalfont Buckinghamshire UK). After detection and quantification of these proteins, each blot was stripped in a Re-blot plus mild antibody stripping solution (CHEMICON, Temecula, CA) for 20 min at room temperature and reprobed for detection of β-tubulin (H-235, Santa Cruz Biotechnology, Inc, Santa Cruz, CA). β-tubulin was used to monitor protein loading among samples. Multiple autoradiographs were obtained using different exposure time, and immunoreactive bands within the linear range of detection were quantified by densitometric scanning using Scion image software (Scion Corp., Frederick, MD).

### Data Analyses

Locomotor activity data are presented as the mean ± standard error of the mean (SEM) number of beam breaks and were analyzed by mixed-factor analyses of variance (ANOVA) with housing condition (EC, IC, or SC) and treatment (saline or nicotine) as between-group factors, and with day and/or time as within-subject factors. Tukey’s post-hoc tests were performed where appropriate. A housing × day × time (3×2×12) mixed factorial ANOVA was used to analyze data from the 2 habituation days, and a housing × time (3×12) factorial ANOVA was conducted on the saline baseline day. The pre-injection habituation part of the experiment was analyzed using a housing × treatment × day × time (3×2×8×12) ANOVA. The effect of repeated nicotine injection on total horizontal activity was analyzed using a housing × treatment × day × time (3×2×8×12) factorial ANOVA, with housing and treatment as between-group factors, and day and time as within-subject factors. Additionally, nicotine-mediated sensitization in EC, IC and SC groups was analyzed by linear regression. To evaluate the effects of repeated nicotine administration on the activity of signaling proteins (total DARPP-32, pDARPP-32 Thr34, pDARPP-32 Thr75, total CREB, and pCREB); separate two-way ANOVAs (housing condition × treatment) were performed on PFC, NAc, and striatum with environment and treatment as between-group factors. Simple effect comparisons were made for post hoc analyses. To determine whether a relationship existed between locomotor activity and immunoreactivity of DARPP-32, pDARPP-32 Thr34, and pDARPP-32 Thr75, separate Pearson correlations were conducted. All statistical analyses were performed using SPSS (standard version 19.0, Chicago, IL). Differences were considered significant at *p*<0.05.

## Results

### Acute Nicotine Administration Regulated DARPP-32 in EC and IC Rats in a Dose-dependent Manner

To determine whether environmental enrichment regulates DARPP-32 signaling in response to nicotine in a dose-dependent manner, we examined the acute effects of nicotine (0.1, 0.3 and 0.8 mg/kg) on pDARPP-32 Thr34, pDARPP-32 Thr75 and total DARPP-32 in PFC, NAc and striatum in EC and IC rats (Table 1). In EC group, nicotine (0.1 mg/kg) increased pDARPP-32 Thr34 levels (42%) in NAc (F_(1,6)_ = 5.2, *p*<0.05), but not in PFC and striatum compared to saline control. However, EC rats treated with nicotine (0.3 mg/kg) showed an increase in pDARPP-32 Thr34 in PFC (45%, F_(1,6)_ = 7.5, *p*<0.05), NAc (116%, F_(1,6)_ = 9.5, p<0.05) and striatum (101%, F_(1,6)_ = 10.2, *p*<0.05). Following 0.8 mg/kg nicotine administration, EC rats had a significant increase of pDARPP-32 Thr34 in PFC (447%, F_(1,6)_ = 12.1, *p*<0.01) and in NAc (59%, F_(1,6)_ = 8.1, *p*<0.01), but not in striatum. In addition, nicotine (0.3 mg/kg) increased pDARPP-32 Thr75 in striatum (58%, F_(1,6)_ = 7.9, *p*<0.01) in EC rats. Acute nicotine had no effects on total DARPP-32 in EC and IC rats. These findings suggest that nicotine dose-dependently increases pDARPP-32 Thr34 levels most prominently in the EC-reared condition and that 0.3 mg/kg acute dose of nicotine is the optimal dose to elicit the most robust changes in pDARPP-32 Thr34 among the measured brain regions of EC and IC rats.

**Table 1:** Ratio of phosphorylated Thr 34 and Thr75 to total DARPP-32 in different brain regions in EC and IC rats after systemic nicotine administration.

	pDARPP-32Thr34/DARPP-32			pDARPP-32Thr75/DARPP-32		
	0.1 mg/kg	0.3 mg/kg	0.8 mg/kg	0.1 mg/kg	0.3 mg/kg	0.8 mg/kg
PFC						
EC-Sal	0.60±0.04	0.69±0.07	0.21±0.07	0.92±0.08	1.19±0.12	1.05±0.08
EC-Nic	0.64±0.08	1.00±0.09*	1.04±0.09*	1.16±0.15	1.15±0.13	1.13±0.11
IC-Sal	0.88±0.09#	0.95±0.08#	0.70±0.09#	1.37±0.16	1.26±0.09	1.05±0.12
IC-Nic	0.95±0.10	1.03±0.11	1.10±0.11	1.08±0.09	0.91±0.08	0.74±0.08
NAC						
EC-Sal	0.76±0.07	0.55±0.09	0.51±0.06	1.02±0.08	1.29±0.15	0.76±0.06
EC-Nic	1.08±0.08*	1.19±0.11*	0.86±0.09*	1.16±0.13	1.23±0.13	0.85±0.08
IC-Sal	1.10±0.11#	0.89±0.09#	1.01±0.12#	1.09±0.15	1.24±0.15	0.97±0.11
IC-Nic	1.11±0.13	1.13±0.12	0.89±0.08	1.43±0.17	1.39±0.14	1.00±0.13
Striatum						
EC-Sal	0.92±0.08	0.68±0.08	0.60±0.09	1.33±0.12	0.82±0.07	0.78±0.09
EC-Nic	0.94±0.07	1.37±0.12*	0.76±0.08	1.47±0.15	1.30±0.13*	0.80±0.09
IC-Sal	1.28±0.06#	1.08±0.09#	1.02±0.11#	1.37±0.17	1.10±0.11	1.09±0.13
IC-Nic	1.18±0.12	1.33±0.12	0.92±0.11	1.40±0.11	0.81±0.09	0.98±0.11

* *p*< 0.05 denotes difference between the nicotine- and saline-treated groups.

# *p*< 0.05 denotes difference between housing groups. n = 10 rats/group.

In the saline control groups, the levels of pDARPP-32 Thr34 were lower in all regions of EC rats relative to IC rats, whereas no significant differences were noted in the levels of pDARPP-32 Thr75 between EC and IC rats. These results suggest environmental enrichment diminishes the basal level of phosphorylated DARPP-32 at Thr34 site.

### Environmental Enrichment Decreased Locomotor Response to Context Novelty

**Figure 1:**
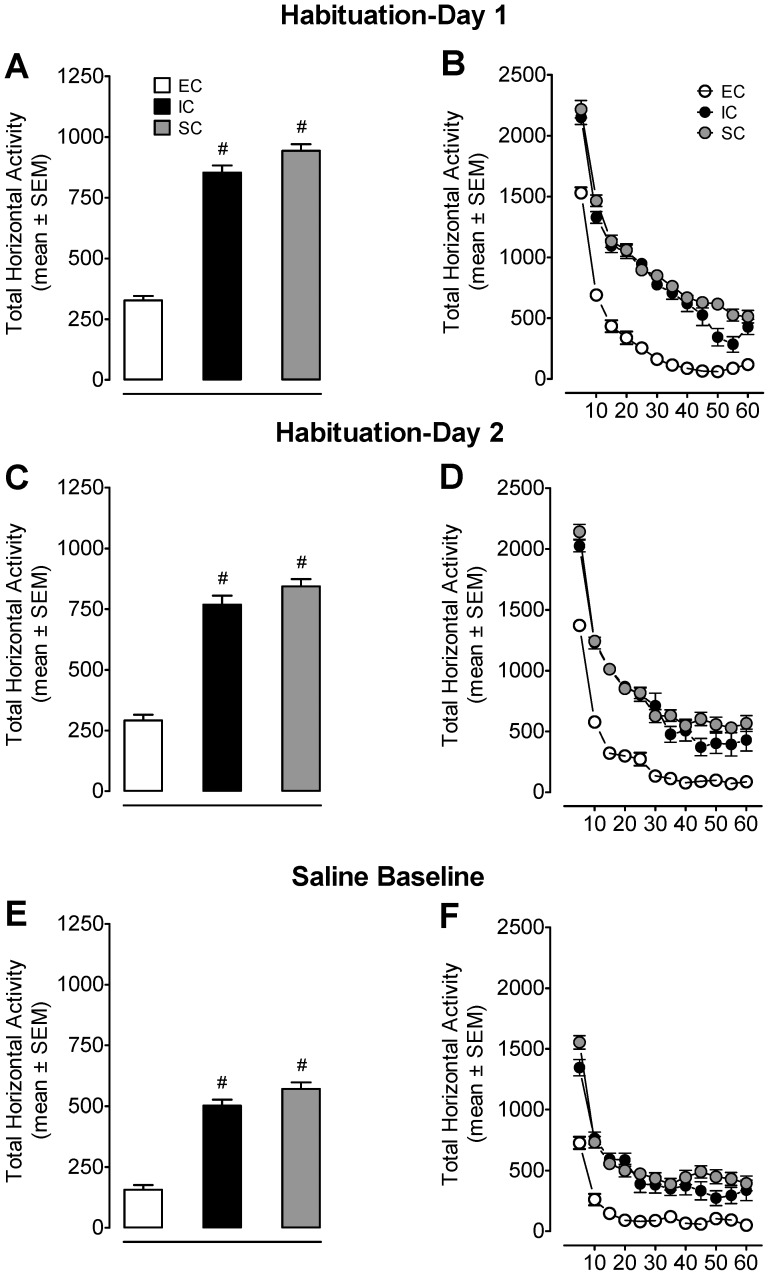
Environmental enrichment diminishes baseline activity during the habituation and saline baseline sessions. Panels A, C, and E show the total horizontal activity (mean ± SEM) across the two 60-min habituation periods and 60-min session post-saline injection. Panels B, D, and F show the time course of the total horizontal activity (mean ± SEM) during each 5-min interval across the two 60-min habituation periods and 60-min session post saline injection. Total horizontal activity revealed a significant effect of housing condition (F_(2, 70)_ = 46.48, *p*<0.001), day (F_(1, 70)_ = 317.41, *p*<0.001) and time (F_(11, 770)_ = 148.57, *p*<0.001), and a significant housing condition × day × time interaction (F_(22, 770)_ = 2.48, p < 0.001). Overall total horizontal activity was lower in EC than in IC or SC (*p*<0.001, Bonferroni *t*-test). # *p*<0.001 denotes difference between EC and IC or SC groups (n = 22–27 rats/group).

Habituation refers to a progressive decrease in locomotor activity following repeated exposure to a particular context. Novelty-elicited locomotor behavior was measured during the two days of habituation to the locomotor chambers for EC, IC, and SC rats. The total activity that occurred for 60 min during the two habituation days is shown in Figure 1 (A–D). A housing condition × day × time ANOVA (3×2×12) revealed main effects of housing condition (*F*_(2, 70)_ = 46.48, *p*<0.001), day (*F*_(1, 70)_ = 317.41, *p*<0.001) and time (*F*_(11, 770)_ = 148.57, *p*<0.001), and a significant housing condition × day × time interaction (*F*_(22, 770)_ = 2.48, *p*<0.001). All animals showed the most activity at the first 10 min of the habituation session, the activity decreased over the following 20 min, and all groups achieved asymptote for the remaining 30 min of the session (Figure 1B and D). EC rats exhibited less locomotor activity than IC and SC rats during the habituation session (*p*<0.01, Bonferroni *t*-test). On the third day, total activity was recorded for all groups after a saline injection to determine baseline activity prior to the induction of the sensitization phase of the experiment (Figure 1E and F). The housing condition × time ANOVA revealed a main effect of housing condition (*F*_(2, 70)_ = 79.02, *p*<0.001) and time (*F*_(11, 770)_ = _108.96_, *p*<0.001), and a housing condition × time interaction (*F*_(22, 770)_ = 4.09, *p*<0.001). In general, all animals showed the most activity during the first 10 min of the saline baseline day, acquired asymptotic levels of activity more quickly, and showed a lower asymptote compared to that of the habituation sessions (Figure 1F). None of the comparisons indicated differences between IC and SC rats (all *ps*>0.05) during the habituation and saline baseline sessions.

### Environmental Enrichment Increased Sensitivity to Nicotine-mediated Locomotor Sensitization

**Figure 2:**
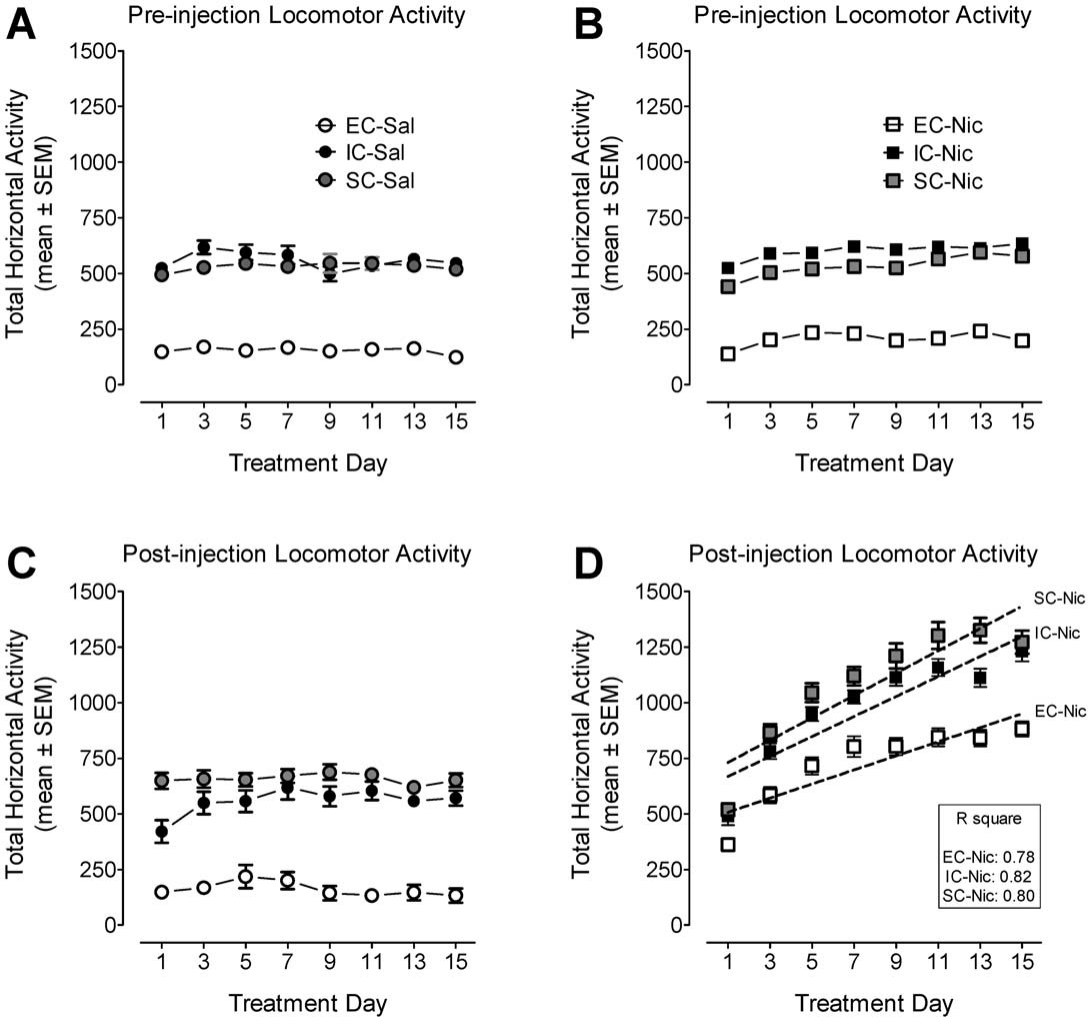
The time-course data during the behavioral sensitization phase. EC, IC or SC rats were administrated nicotine (Nic, 0.35 mg/kg, s.c.) or saline (Sal) on Days 1–15. Panels A and B show the total horizontal activity (mean ± SEM) in saline control and nicotine-treated groups during the 30 min pre-injection habituation period. Panels C and D shows the total horizontal activity (mean ± SEM) during the 60 min following saline or nicotine injection with robust behavioral sensitization observed under all housing conditions (panel D). Total horizontal activity after a subcutaneous injection of saline or nicotine revealed a significant effect of housing condition (F_(2, 67)_ = 104.29, *p*<0.001), treatment (F_(1, 67)_ = 333.57, *p*<0.001) and day (F_(7, 469)_ = 139.97, *p*<0.001). A significant interaction of housing condition × treatment × day (F_(14, 469)_ = 2.76, *p*<0.05) and treatment × day (F_(7, 469)_ = 115.42, *p*<0.05) were found. n = 12 rats/group.

All animals were placed into locomotor chambers for 30 min prior to the activity measurement to produce within-session habituation of activity in response to the context prior to nicotine or saline injection. Total activity in saline control and nicotine-treated group during the 30 min habituation period across the 15-day treatment was recorded as shown in Figure 2A and B. A mixed-factor housing condition × treatment × day × time ANOVA (3×2×8×6) revealed main effects of housing condition (F_(2, 67)_ = 333.90, *p*<0.001), treatment (F_(1, 67)_ = 5.16, *p*<0.05), day (F_(7, 469)_ = 298.97, *p*<0.001), time (F_(5, 335)_ = 47.77, *p*<0.001) and a significant day × housing condition interaction (F_(14, 469)_ = 7.75, *p*<0.001). There were no main effects of treatment and interactions containing this factor. EC rats exhibited less locomotor activity than did IC and SC rats across the pre-injection habituation sessions (*p*<0.01, Bonferroni *t*-test).

To determine whether differential housing environments alter nicotine-mediated locomotor sensitization, we analyzed locomotor activity during daily administration of nicotine (0.35 mg/kg) or saline during Days 1–15 for EC, IC, and SC rats (Figure 2C and D). A housing condition × treatment × day ANOVA analysis revealed significant main effects of housing condition (F_(2, 67)_ = 104.29, *p*<0.001), treatment (F_(1, 67)_ = 333.57, *p*<0.001) and day (F_(7, 469)_ = 139.97, *p*<0.001). A significant housing condition × treatment × day interaction (F_(14, 469)_ = 2.76, *p*<0.05) was found. As shown in Figure 2D, linear regression analyses revealed that nicotine-induced locomotor activity gradually increased across treatment days under all housing environment conditions: EC (F_(1, 6)_ = 20.97, *p*<0.01), IC (F_(1, 6)_ = 27.94, *p*<0.01) and SC (F_(1, 6)_ = 33.11, *p*<0.01). There was no significant difference across the slopes of the three housing groups, but a significant difference in intercepts or elevations was noted (F_(2, 20)_ = 22.48, *p*<0.001). Thus, nicotine injection produced a robust behavioral sensitization in EC, IC and SC rats.

Based on the significant three-way interaction, separate two-factor ANOVAs were conducted. In saline-treated groups, a housing condition × day ANOVA analysis revealed significant main effects of housing condition (F_(2, 34)_ = 80.77, *p*<0.001) and day (F_(7, 238)_ = 2.52, *p*<0.05). Also, a significant housing condition × day interaction (F_(14, 238)_ = 1.84, *p*<0.001) was found, indicating repeated overall difference in total activity among the different housing groups. Post hoc tests (Bonferroni *t*-test) showed that EC rats were significantly less active than were IC or SC rats across treatment days (*p*<0.001), but no difference between IC and SC rats was found (*p*>0.05). In nicotine-treated groups, the two-way ANOVA analysis revealed significant main effects of housing condition (F_(2, 33)_ = 32.57, *p*<0.001) and day (F_(7, 231)_ = 243.45, *p*<0.001), but post hoc tests showed no difference between the IC and SC groups. There was a significant housing condition × day interaction (F_(14, 231)_ = 5.51, *p*<0.001), suggesting EC rats have lower nicotine-mediated locomotor activity across treatment days. Overall, all rats treated with nicotine displayed greater locomotor activity on Days 3–15 than on Day 1 (*ps*<0.05).

**Figure 3:**
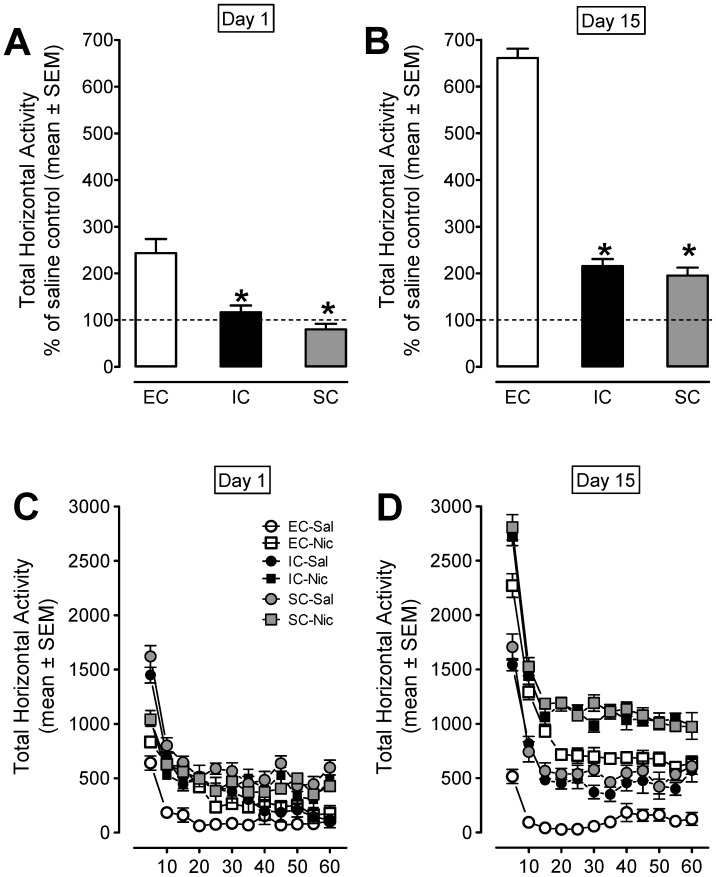
The time-course data for total horizontal activity during day 1 and day 15 of the behavioral sensitization phase. Panels A and B show the total horizontal activity (mean ± SEM) across the 60-min session. Data are presented as percent of their saline controls for respective housing condition. Panels C and D show the time course of the total horizontal activity (mean ± SEM) during each 5-min interval. * *p*<0.05 between EC and IC or SC groups. n = 12 rats/group.

The time course effect of nicotine in EC, IC and SC rats on Days 1 and 15 is illustrated in Figure 3. A housing condition × treatment × day × time ANOVA revealed significant main effects of housing condition (F_(2, 67)_ = 108.16, *p*<0.001), treatment (F_(1, 67)_ = 243.44, *p*<0.001), day (F_(1, 67)_ = 209.67, *p*<0.001) and time (F_(11, 737)_ = 231.97, *p*<0.001). In addition, there was a significant housing condition × treatment × day interaction (F_(2, 67)_ = 3.66; *p*<0.05). When the data were expressed as a percentage change relative to the respective saline controls, on Day 1 acute nicotine increased locomotor activity (243%) in EC (*p*<0.001, Bonferroni *t*-test) and (116%) in IC, but decreased activity (20%) in SC rats (*p*<0.05, Figure 3A). On day 15, nicotine produced hyperactivity in EC rats (661%), IC (215%) and SC (195%) relative to their respective saline control. In addition, compared to Day 1, repeated nicotine administration elevated activity to a greater extent in EC (271%), IC (185%), and SC (243%) rats on Day 15, suggesting that the EC rats exhibit increased sensitivity to behavioral sensitization. The time course data in these rats on Day 1 and Day 15 are illustrated in Figure 3C and D. *Post hoc* tests showed that all rats had greater activity during the first 10 min and that nicotine-treated rats exhibited higher activity counts throughout the remainder of the 60 min.

### Repeated Nicotine Administration Differentially Regulated Phosphorylation of DARPP-32 Protein in EC, IC, and SC Rats

**Figure 4:**
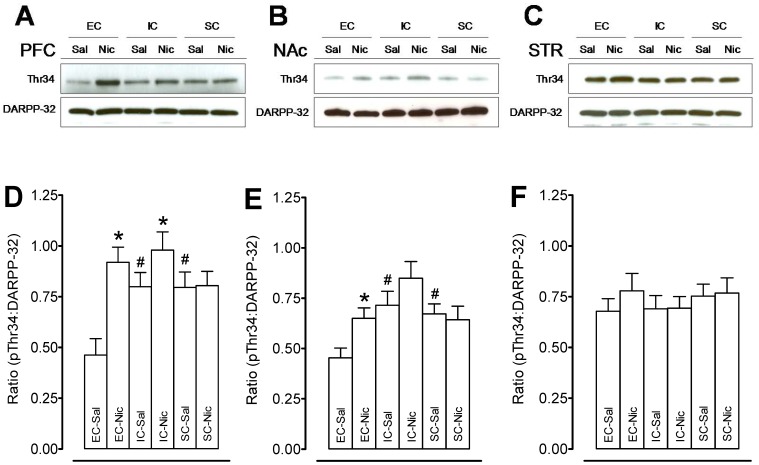
Levels of pDARPP-32 Thr34 and total DARPP-32 in prefrontal cortex, nucleus accumbens and striatum in EC, IC, and SC rats. Top panels show representative immunoblots of pDARPP-32 Thr34 and total DARPP-32 in prefrontal cortex (PFC, A), nucleus accumbens (NAc, B) and striatum (STR, C) in nicotine or saline-treated rats. Bottom panels show the ratio of levels of pDARPP-32 Thr34 to total DARPP-32 in PFC (D), NAc (E) and STR (F) of nicotine (0.35 mg/kg) or saline-treated rats. Data are presented as the percentage of pDARPP-32 Thr34 to total DARPP32 densitometry values of immunoreactivity. Histobars represent means and bars are SEM. Total DARPP-32 and pDARPP-32 Thr34 were run at the same time with the same loading amount of protein. No statistically significant differences were found with total DARPP-32 levels in any of the groups. *p<0.05 denotes difference between the nicotine- and saline-treated groups. #*p*<0.05 denotes difference between housing groups. n = 10 rats/group.

The pathway of DARPP-32 signaling is crucial for neuroadaptation in response to repeated nicotine administration [29], [35]. Therefore, we determined whether DARPP-32 levels and phosphorylation states were altered in parallel with the differential behavioral response to nicotine in EC, IC, and SC rats. The levels of DARPP-32 and pDARPP-32 Thr34 in PFC, NAc and striatum of the EC, IC, and SC rats, whose behavioral measurements were applied, are illustrated in Figure 4. No differences were found in total DARPP-32 and pDARPP-32 Thr75 in PFC, NAc, and striatum among the three housing conditions (data not shown).

A two-way ANOVA on the levels of pDARPP-32 Thr34 in PFC of the rats treated with saline or nicotine revealed a main effect of housing condition (F_(2, 53)_ = 3.43, *p*<0.05) and a trend of housing × treatment interaction (F_(2, 53)_ = 2.87, *p* =  0.06), but no significant effect of treatment (Figure 4 A and D). In saline controls, one-way ANOVAs revealed the level of pDARPP-32 Thr34 in PFC in the EC group was lower than that in both the IC and SC groups (*p*<0.01), but no difference between the IC and SC groups was found. Following repeated nicotine administration, the level of pDARPP-32 Thr34 in PFC was increased in the EC (50±2.8%, F_(1, 16)_ = 8.32, *p*<0.05) and IC (18±0.2%, F_(1, 16)_ = 3.12, *p*<0.05) compared to the respective saline controls. No difference between SC-Sal and SC-Nic groups was found. In NAc, two-way ANOVA on the levels of pDARPP-32 Thr34 revealed a main effect of housing condition (F_(2, 53)_ = 4.21, *p*<0.05), and no significant effect of treatment or their interaction (Figure 4B and E). EC rats exhibited decreased basal pDARPP-32 Thr34 level compared to the IC and SC rats (47±3.5%and 39±2.8%, respectively). Repeated nicotine significantly increased pDARPP-32 Thr34 level in the EC rats (30±2.1%, *p*<0.05), but not in IC and SC rats (*ps*>0.05). In striatum, no difference in pDARPP-32 Thr34 level was found among EC, IC and SC rats with nicotine or saline injection (Figure 4C and F).

### Repeated Nicotine Administration Differentially Regulated Phosphorylation of CREB in EC, IC, and SC Rats

**Figure 5:**
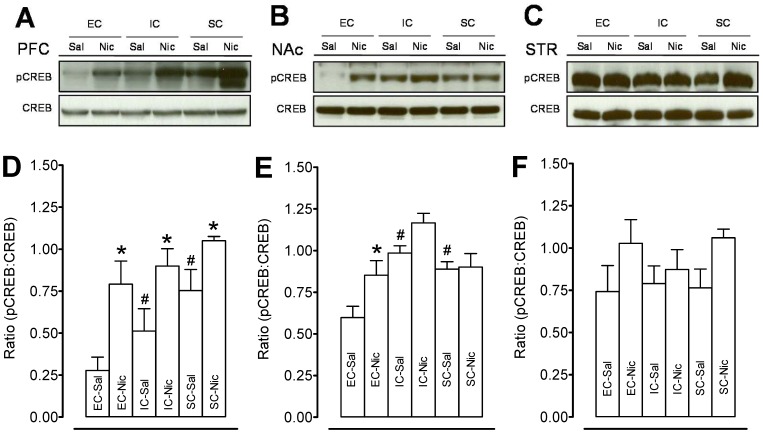
Levels of pCREB and total CREB in prefrontal cortex, nucleus accumbens, and striatum in EC, IC, and SC rats. Top panels show representative Western blots of pCREB and CREB in prefrontal cortex (PFC, A), nucleus accumbens (NAc, B) and striatum (STR, C). Bottom panels show the ratio of phosphorylated CREB levels to total CREB in PFC (D), NAc (E) and STR (F) of nicotine (0.35 mg/kg) or saline-treated rats. Data are presented as the percentage of pCREB to total CREB densitometry values of immunoreactivity. Histobars represent means and bars are SEM. Total CREB and pCREB were run at the same time with the same loading amount of protein. No statistically significant differences were found with total CREB levels in any of the groups. *p<0.05 denotes difference between the nicotine- and saline-treated groups. #*p*<0.05 denotes difference between housing groups. n = 10 rats/group.

We examined whether environmental enrichment changed CREB and pCREB in the PFC, NAc, and striatum in the EC, IC, and SC groups. As shown in Figure 5, no significant differences in total CREB were found in these regions among the groups. With respect to the ratio of pCREB /CREB in the PFC (Figure 5A and D), a main effect of housing condition (F_(2, 18)_ = 21.22, p < 0.001) and treatment (F_(1, 18)_ = 98.64, p < 0.001), and a significant housing condition × treatment interaction (F_(1, 18)_ = 4.69, p < 0.05) were found. *Post hoc* analysis revealed that the ratio of pCREB /CREB was lower in EC-Sal than in IC-Sal (F_(1, 6)_ = 11.44, *p*< 0.05) and SC-Sal (F_(1, 6)_ = 58.00, *p*<0.001), indicating that environmental enrichment decreases the basal levels of pCREB. Repeated nicotine administration significantly increased pCREB levels in PFC of EC (185±16%, F_(1, 6)_ = 88.57, *p*<0.001), IC (75±12%, F_(1, 6)_ = 29.57, *p*<0.01), and SC (39±2.9%, F_(1, 6)_ = 21.35, *p*<0.01) compared to the respective saline control groups.

With respect to the ratio of pCREB /CREB in the NAC (Figure 5B and E), a main effect of housing condition (F_(2, 18)_ = 9.34, *p*<0.05) was found. There was no significant effect of treatment and their interaction. The ratio of pCREB /CREB was lower in EC-Sal than in IC-Sal (F_(1, 6)_ = 5.92, *p*<0.05) and SC-Sal (F_(1, 6)_ = 4.89, *p*<0.05). Repeated nicotine administration increased the level of pCREB in EC-Nic rats (42±3.0%, F_(1, 6)_ = 6.71, *p*<0.05) but not in IC-Nic and SC-Nic rats. In striatum, no differences in pCREB and total CREB levels were found among the EC, IC and SC rats with nicotine or saline injection (Figure 5C and F).

### Alterations of Locomotor Behavior were Associated with pDARPP-32 Thr34 Levels in PFC

**Figure 6:**
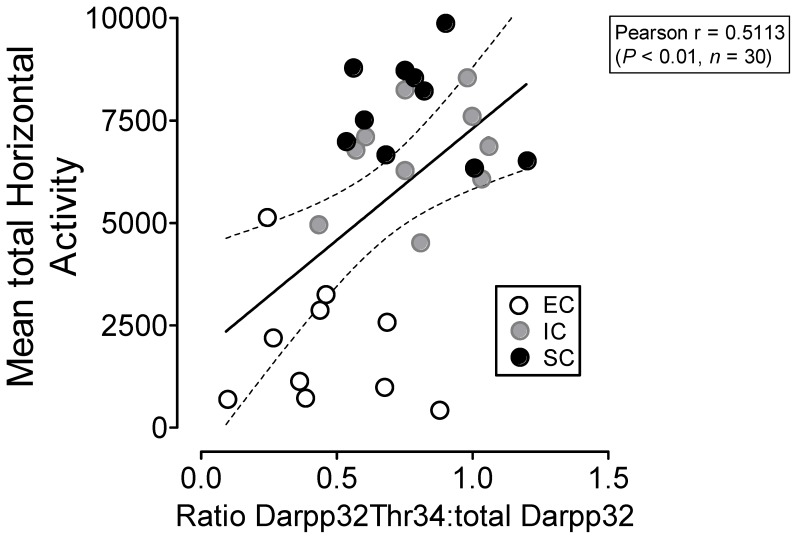
Correlations of the phosphorylation state of DARPP-32 at Thr34 in PFC with locomotor activity for EC, IC, and SC saline-treated rats. Locomotor activity counts were collected from behavioral testing on day 15. Total horizontal activity is presented as mean values of each rat within the 60-min session. Phosphorylation levels of DARPP-32 at Thr34 are presented as the percentage of pDARPP-32 Thr34 to total DARPP-32 densitometry values of immunoreactivity. Dashed lines represent the 95% confidence interval of the linear regression fit (solid line). n = 10 rats/group.

To determine whether the basal level of DARPP-32 activity was associated with the results of behavior tests, the correlation of locomotor activity and DARPP-32 activity was examined. Figure 6 illustrated the correlations of immunoblot densities of pDARPP-32 Thr34 in PFC of all saline control rats and their respective locomotor counts collected from last day (Day 15, Session 8) of behavioral tests. There were no correlations regarding total horizontal activity and total DARPP-32 protein levels (*p* = 0.69, Pearson *r* = −0.08, data not shown) or pDARPP-32 Thr75 protein levels (*p* = 0.79, Pearson *r* = 0.051, data not shown). However, pDARPP-32 Thr34 protein levels were correlated positively with mean total horizontal activity (Fig. 6, *p*<0.01, Pearson *r* = 0.51). Correlations were also examined in the nicotine-treated rats; however, no statistically significant correlations were found (data not shown). Together, these results suggest that pDARPP-32 Thr34 levels in the PFC are responsible for EC reductions in locomotor basal levels and possibly involved in repeated nicotine-induced locomotor sensitization.

## Discussion

The current findings demonstrate that an enriched housing environment alters the levels of phosphorylated DARPP-32 and CREB under control conditions (i.e. after a saline injection) and following nicotine administration. Specifically, the effects of enrichment on activity of DARPP-32 and CREB are robustly found in PFC relative to NAc and striatum. The fact that the basal phosphorylation state of DARPP-32 at Thr34 site in PFC is positively correlated with locomotor activity in EC, IC, and SC rats under saline control conditions, suggests that the PFC DARPP-32 phosphorylation at Thr34 may play an important role in enriched environment-induced changes in locomotion. Acute nicotine injection produces increased levels of pDARPP-32 Thr34 in EC rats in a dose-dependent manner. Although repeated nicotine administration produced locomotor sensitization in EC, IC, and SC rats, when the nicotine-mediated activity was expressed as a percent change from the respective saline controls EC rats displayed greater sensitization to nicotine than IC and SC rats. In addition, the magnitude of change from saline control in nicotine-induced enhancement of pDARPP-32 Thr34 and pCREB in PFC was strikingly increased in EC rats relative to IC and SC rats, indicating a potential mechanism through which environmental enrichment could reduce locomotor effects of nicotine. Together, the findings demonstrate a novel role of PFC pDARPP-32 Thr34 in enriched environment-induced neuroplasticity and behavioral changes associated with repeated nicotine administration.

Relative to the IC condition, the complexity of an enriched environment paradigm comprises multiple components: a large space, physical exercise, novel objects, and social cohorts. We acknowledge that one limitation of the current study is that the results do not address which of these components, or combination of components, is specifically responsible for the environmental-induced behavioral and neurochemical changes. To examine this issue completely, several variations of “control” conditions would be needed, including manipulations to study the effects of cages size, numbers of social partners, presence of novel objects, amount of exercise, and so on, which is beyond the scope of the current study. In this study, we chose a social control condition with only one other animal in a small cage because this represents the NIH standard housing condition, which is the most typical housing condition used across various laboratories and allows for cross-comparisons with results published in the literature. However, some studies have used other controls, such as single-housed animals with novel objects or social caged animals (n = 8–10 per cage) without novel objects. For example, in a recent study [36], an SC condition (using the larger EC cages with no toys), and a novelty condition (NC, using the isolated IC cages with two plastic toys rotated daily) were used as control conditions for the EC group. When the escalation of cocaine (0.1 mg/kg) was examined in a self-administration paradigm, only NC and IC rats showed escalation [36], suggesting that social cohorts may be a primary factor in the behavioral effects of an enriched environment. In addition, the effect of exercise has been shown to have dramatic effects on reducing drug-taking behavior as well as influencing neurochemical changes [37]–[40]. Moreover, when only novelty was used to distinguish between enriched and standard environments (same size cage and number of cohorts), enrichment in mice eliminated both behavioral sensitization and conditioned place preference to cocaine [41], suggesting novelty acts as the main neuroprotective factor. The findings from our current study, as well as the reported above, suggest that the effects of each enrichment component on behavioral and neurochemical changes is an interesting topic for future study.

The most important finding from the study is that EC rats show diminished basal levels of pDARPP-32 Thr34 in PFC and NAc relative to IC and SC rats under saline control conditions, indicating that PKA-mediated Thr34 pathway is quite sensitive to enrichment-induced plasticity. The Thr34 phosphorylation levels are regulated by both dopaminergic and glutamatergic receptor stimulation signaling [27]. D1 receptor stimulation results in phosphorylation at Thr34, whereas activation of D2 receptor of DA and N-methyl-D-aspartate (NMDA) glutamate receptor results in the dephosphorylation of Thr34 [42], [43]. Compared to IC rats, EC rats exhibit decreased expression and functioning of D1 receptors in PFC [20], which may cause lower dopaminergic tone under basal condition in EC rats compared to IC rats [18], [19], [44]. On the other hand, environmental enrichment enhances excitatory glutamatergic synaptic transmission in cortex [45], which may induce dephosphorylation of Thr34 by activation NMDA receptors. Therefore, an altered balance of D1/cAMP/PKA signaling and glutamate transmission in PFC may cause a difference in the basal levels of pDARPP-32 Thr34 between EC and IC rats.

Acute nicotine regulated the phosphorylation levels of DARPP-32 at Thr34 and Thr75 sites in a dose-dependent manner. While acute nicotine (0.1 mg/kg) only increased Thr34 levels in NAc of EC rats and had no effects on Thr75, the high dose of nicotine (0.8 mg/kg) increased Thr34 levels in PFC and NAc levels in EC rats. However, nicotine (0.3 mg/kg) obviously increased Thr34 levels in all regions and Thr75 levels in striatum in EC rats, suggesting an enhanced maximal action of nicotine on the phosphorylation of DARPP-32. Under *in vitro* conditions (mouse striatal slices), nicotine at a low concentration decreases Thr34 levels, whereas nicotine at a high concentration increases Thr34 and decreases Thr75 levels [46], [47]. Although systemic acute nicotine (0.8 mg/kg, s.c.) increased phosphorylation of both Thr34 and Thr75 in mouse striatum 15 min after injection [48], another report showed no effects on Thr34 and Thr75 in striatum and NAc of rats 20 min after a single injection of nicotine (0.35 mg/kg, s.c.) [29]. Thus, nicotine-mediated regulation of DARPP-32 activity is largely dependent on the species, dosage, route of administration, and the time needed to harvest brains. Importantly, in the current study, nicotine produced increased pDARPP-32 Thr34 in EC rats, which may be caused by an intrinsic difference in basal levels of Thr34 between EC and IC rats. The action of nicotine on Thr34 is regulated by activation of the D1 receptor-mediated PKA pathway [47], and this cascade contributes to nicotine-induced motivation [49]. The current results show that the behaviorally-relevant dose of nicotine (0.35 mg/kg) produce hyperactivity in EC and IC rats, but hypoactivity in SC rats on Day 1. Thus, the differential regulatory effects on pDARPP-32 Thr34 levels in response to acute nicotine in EC and IC rats may play a role in the differential locomotor response to nicotine between EC and IC rats.

Repeated nicotine administration eliminated the basal difference in pDARPP-32 Thr34 observed between the EC and IC rats and increased pDARPP-32 Thr34 in the PFC of EC rats relative to IC rats. Nicotine also elicited Thr34 increases in the NAc of EC rats, despite being not as robust as in the PFC. This implicates that the processes mediating the lower basal levels of pDARPP-32 Thr34 in the PFC of EC rats do not prevent repeated nicotine stimulation from regulating DARPP-32 signaling. Rather, compared to their respective saline controls, the magnitude of change in nicotine-induced Thr34 level in PFC is greater in EC than in IC and SC rats. DA D1 receptor activation has been demonstrated to increase Thr34 levels [50] and it is possible that the greater increase in nicotine-induced Thr34 levels in PFC of EC rats may represent a compensatory D1 receptor-mediated down-regulation in response to nicotine-stimulated enhancement of DA transmission. While the current results show that no effects of repeated nicotine stimulation on pDARPP-32 Thr34 levels in striatum were found in EC, IC, and SC rats, a previous study has shown that repeated nicotine (0.35 mg/kg) produces a clear increase in pDARPP-32 Thr34 in the dorsal striatum of rats [29]. One possibility for the discrepancy could be due to the different methods of sample preparations, for example, the entire rat head 15 min after last nicotine injection was immediately frozen in chilled 2-methyl butane and brains were later dissected [29], whereas in the current study, brain regions were immediately dissected and sonicated in sample buffer as described previously [32]. Similarly, our previous study using the *in vivo* voltammetry assay has demonstrated that basal DA clearance in medial PFC is lower in EC rats than IC rats under saline control condition, whereas systemic acute nicotine injection only increases the DA clearance in EC rats, but not in IC rats [21]. The decrease in DA clearance in medial PFC of EC rats is the result of reduced DA transporter surface expression [51]. Given that DA induced internalization of D1 receptors in HEK293 cells [52], it is possible that a greater proportion of D1 receptors in IC rats are internalized by nicotine-induced DA release resulting in a reduced molecular response to nicotine in IC rats. Although little evidence shows that enrichment-induced manipulations of DA signaling within the mesolimbic circuit attenuate nicotine-mediated sensitization, the current results demonstrate a complex regulatory mechanism underlying differential molecular effects on nicotine between EC and IC rats. Thus, enrichment-induced neuroplasticity showing decreases in basal Thr34 levels may allow EC rats to have an increased molecular response to nicotine.

In addition to DARPP-32, this study also determined the effects of enrichment on CREB activity following repeated saline and nicotine administration. Enrichment-mediated changes in phosphorylated CREB at Ser133 are in parallel with the changes of pDARPP-32 Thr34 levels, which are associated with PKA activation [24], [26]. Basal pCREB levels were lower in PFC and NAc of EC rats than IC and SC rats in the saline control group, which is in agreement with a previous report showing lower levels of pCREB in the NAc of EC rats [53]. However, the magnitude of change in nicotine-induced increase in pCREB in PFC and NAc was greater in EC rats than IC and SC rats. At the cellular levels, phosphorylation of DARPP-32 at Thr34 by PKA converts to a potent inhibitor of the protein phosphatase-1, which controls the phosphorylation state of CREB [27], [54]. Activation of D1 receptors has been shown to increase the levels of phosphorylation of Thr34 and pCREB in rat PFC [55]. Thus, enriched environment-induced changes in the levels of both pDARPP-32 Thr34 and pCREB in PFC is consistent with a regulatory role of the D1/cAMP/PKA signaling pathway.

Our results demonstrated that EC rats had greater nicotine-mediated increases in pDARPP-32 Thr34 and pCREB, which were robustly found in the PFC relative to the NAc, and striatum. PFC, specifically prelimbic and infralimbic cortices as well as nucleus accumbens have been implicated in addiction, extinction, and relapse and have been hypothesized to constitute a series circuit underlying behavioral changes in response to psychostimulants [56]. The prelimbic cortex is responsible for maintaining the relationship between goal and actions, along with compulsive responding [57], [58], whereas the infralimbic cortex is responsible for impulsive action and for controlling the inhibition on goal-directed actions [38], [59]. Several lines of evidence have suggested that DA and glutamate inputs play a crucial role in modulating the activity of the PFC in response to psychostimulants [60]–[64]. Previous studies have demonstrated that drug-naïve EC rats exhibit less synaptic DA levels [18], [44], diminished DA transporter function [17], [51], and decreased expression and function of the DA D1 receptor [20], compared to IC rats. These changes are primarily found in PFC but not NAc and striatum, indicating that PFC is a predominant region involved in enriched environment-induced neuroplasticity. In a parallel experiment, we found no changes in immunoreactivity of NMDA glutamate receptors (NR1, NR2A and NR2B) in PFC, NAc and striatum among EC, IC, and SC rats following repeated saline or nicotine injections (data not shown). In fact, it has been shown that activation of D1 receptors increases NMDA-mediated PFC pyramidal cell excitability through postsynaptic PKA activity [65]. Therefore, these findings support the view that the DA/D1 receptor/PKA-regulated signaling pathway may contribute to the mechanism of environment-dependent plasticity of the PFC. In addition, the PFC during development exhibits dynamic change in gray and white matter proceeding [66], which may associated with increased dopaminergic innervations in PFC in adolescence, a period of high risk for drug vulnerability [67], [68]. Together, the current study demonstrates that the enriched environment-induced alteration of phosphorylated DARPP-32 and CREB may contribute to neuroadaptation of mesocorticolimbic regulatory circuitry, leading to resistant-like behavior to psychostimulants. A full understanding of the neurobehavioral mechanisms underlying enriched environment-induced resistance to psychostimulants is important for targeting those individuals most vulnerable to psychostimulant abuse and may aid the discovery of novel pharmacological treatments for drug abuse.

The baseline locomotor activity in all saline control animals was correlated positively with the respective basal levels of pDARPP-32 Thr34 in PFC, but not Thr75 and total DARPP-32, suggesting that the basal phosphorylation state of Thr34 in PFC is associated with changes in basal locomotor activity in housing conditions. Notably, because EC rats have lower baseline levels of ambulatory activity relative to IC and SC rats, it is important to point out how to express environment-dependent differences in drug effects when the saline-treated control groups differ. In the current study, the nicotine-mediated locomotor activities in EC, IC, and SC groups on Days 1 and 15 were expressed as percent changes from the respective saline controls; EC rats actually showed greater locomotor sensitization to nicotine than IC and SC rats. However, when the nicotine-induced activities in the three housing groups were expressed as absolute values, EC rats had less sensitivity to nicotine-induced sensitization than that in IC and SC rats [16], [31]. This difference in data presentation could complicate the interpretation of locomotor effects of nicotine on enriched environment-induced changes in nicotine-mediated motivation. Since baseline differences in behavior represent intrinsic differences between EC and IC rats, these results highlight the importance of considering the multiple assessments of the data when attempting to depict potential environment-dependent differences in various drug effects. EC rats showed enhanced sensitization to nicotine, which was accompanied by a more robust increase of nicotine-mediated pDARPP-32 Thr34 levels compared to IC or SC rats. Therefore, our molecular findings showing an increase of nicotine-mediated pDARPP-32 Thr34 levels in EC rats may further support this notion of increased behavioral responsiveness to repeated nicotine stimulation in EC rats. However, the nicotine-mediated activity was not correlated with the levels of pDARPP-32 Thr34 in any region examined. One possibility is that nicotine elevated pDARPP-32 Thr34 levels in EC and IC rats to its maximum potential thereby causing a ceiling effect on nicotine-mediated pDARPP-32 Thr34. Evidence suggests that DARPP-32 and its phosphorylation at Thr34 have an inhibitory role in spontaneous locomotor activity, morphine- or cocaine-induced locomotor sensitization and nicotine-induced motor depression in mice [48], [69]–[71]. Given the important role of the phosphorylation at Thr34 of DARPP-32 in stimulant self-administration [72], the current results may also have relevance to environmental enrichment-induced potential resistance to drug-self-administration. One caveat is that although behavioral sensitization is a sensitive measure for the influence of psychostimulants on the mesocorticolimbic system [73], it does not measure drug reward. Thus, the neurobiological changes found in the behavioral sensitization model, in this study, may not be completely recapitulated in human smokers. On the other hand, direct, placebo-controlled evidence for behavioral sensitization has been documented in humans [74] and a growing number of findings demonstrate that drug sensitization has long-lasting effects on behavior and cognition beyond mere changes in locomotor activity and drug taking [75], [76]. There have also many links showing that sensitization may be responsible for the initiation and maintenance of psychostimulant intake in a more complete behavioral model of addiction, self-administration [77], [78]. Therefore, the current findings, at least in part, infer that cigarette smoking in humans would produce alterations in motivated behavior due to the changes in signaling proteins within the mesocorticolimbic DA system. In fact, EC rats display altered self-administration behavior to both amphetamine and cocaine [36], [79]. We speculate that intake of nicotine in EC rats might also differ in the self-administration model of drug addiction. To determine the role of PFC pDARPP-32 Thr34 in nicotine self-administration in EC rats is an essential task in our future study.

In conclusion, the current study has begun to identify the neurobiological mechanism of enriched environment-induced alterations in DARPP-32 and CREB activity on altering sensitivity to the locomotor effects of nicotine. More specifically, the basal levels of PFC pDARPP-32 Thr34 are correlated positively with the baseline locomotor activity in rats housed in different housing conditions. Future studies will investigate whether manipulation of prefrontal cortical DARPP-32 phosphorylation attenuates the difference in basal and nicotine-mediated behavior, which will allow us to better understand the neurobiological basis of environmental enrichment in potential resistance to the motivational responses to psychostimulants.
